# On Weyl products and uniform distribution modulo one

**DOI:** 10.1007/s00605-017-1100-8

**Published:** 2017-09-26

**Authors:** Christoph Aistleitner, Gerhard Larcher, Friedrich Pillichshammer, Sumaia Saad Eddin, Robert F. Tichy

**Affiliations:** 1 Institute for Analysis and Number Theory, Graz University of Technology, Graz, Austria; 2 Department of Financial Mathematics and Applied Number Theory, Johannes Kepler University Linz, Linz, Austria

**Keywords:** Trigonometric product, Star-discrepancy, Kronecker sequence, van der Corput sequence, 11K06, 11K31, 11L15

## Abstract

In the present paper we study the asymptotic behavior of trigonometric products of the form $\prod _{k=1}^N 2 \sin (\pi x_k)$ for $N \rightarrow \infty$, where the numbers $\omega =(x_k)_{k=1}^N$ are evenly distributed in the unit interval [0, 1]. The main result are matching lower and upper bounds for such products in terms of the star-discrepancy of the underlying points $\omega$, thereby improving earlier results obtained by Hlawka (Number theory and analysis (Papers in Honor of Edmund Landau, Plenum, New York), 97–118, 1969). Furthermore, we consider the special cases when the points $\omega$ are the initial segment of a Kronecker or van der Corput sequences The paper concludes with some probabilistic analogues.

## Introduction and statement of the results

Let *f* be a function $f:[0,1] \mapsto \mathbb {R}_{0}^{+}$ and $(x_{k})_{k \ge 1}$ be a sequence of numbers in the unit interval. Much work was done on analyzing so-called *Weyl sums* of the form $S_{N} := \sum ^{N}_{k=1} f(x_{k})$, and on the convergence behavior of $\frac{1}{N} S_{N}$ to $\int ^{1}_{0} f(x) \,\mathrm{d}x$. See for example [8, 17, 36, 41]. It is the aim of this paper to propagate the analysis of corresponding “*Weyl products*”

$$\begin{aligned} P_{N} := \prod ^{N}_{k=1} f(x_{k}), \end{aligned}$$

in particular with respect to their asymptotic behavior for $N \rightarrow \infty$.

Note that, formally, studying products $P_{N}$ in fact is just a special case of studying $S_{N}$, since

$$\begin{aligned} \log P_{N} = \sum ^{N}_{k=1} \log f(x_k), \end{aligned}$$

unless $f(x)= 0$ for some $x \in [0,1]$. Thus we will concentrate on functions *f* for which $f(0) =0$ (and possibly also $f(1) = 0)$.

Assuming an even distribution of the sequence $(x_{k})_{k \ge 1}$, one expects $\frac{1}{N} \sum ^{N}_{k=1} \log f(x_k)$ to tend to the integral $\int ^{1}_{0} \log f(x) \,\mathrm{d}x$ if this exists. That means, very roughly, that we expect

$$\begin{aligned} \prod ^{N}_{k=1} f(x_{k}) \approx \left( \mathrm{e}^{\int ^{1}_{0} \log f(x) \,\mathrm{d}x}\right) ^{N}, \end{aligned}$$

which we can rewrite as

$$\begin{aligned} \prod ^{N}_{k=1} S_{f} \, f(x_k) \approx 1, \quad \text {where} \quad S_{f} := \mathrm{e}^{- \int ^{1}_{0} \log f(x) \,\mathrm{d}x}. \end{aligned}$$

Hence it makes sense to study the asymptotic behavior of the normalized product

$$\begin{aligned} \prod ^{N}_{k=1} S_{f} f(x_k) \ ~ \text{ rather } \text{ than } \ ~\prod ^{N}_{k=1} f(x_k). \end{aligned}$$

A special example of such products played an important role in [1] in the context of pseudorandomness properties of the Thue–Morse sequence, where *lacunary* trigonometric products of the form

$$\begin{aligned} \prod ^{N}_{k=1} 2 \sin (\pi 2^{k} \alpha ) \end{aligned}$$

for $\alpha \in \mathbb {R}$ were analyzed. (Note that $\int _0^1 \log \sin (\pi x) \mathrm{d} x = - \log 2$, hence the normalization factor 2 in this case.)

It was shown there that for almost all $\alpha$ and all $\varepsilon >0$ we have

1 $$\begin{aligned} \prod ^{N}_{k=1} |2 \sin ( \pi 2^{k} \alpha )| \le \exp \left( (\pi + \varepsilon ) \sqrt{N \log \log N}\right) \end{aligned}$$

for all sufficiently large *N* and

2 $$\begin{aligned} \prod ^{N}_{k=1} |2 \sin ( \pi 2^{k} \alpha ) | \ge \exp \left( (\pi -\varepsilon )\sqrt{N \log \log N}\right) \end{aligned}$$

for infinitely many *N*.

In the present paper we restrict ourselves to $f(x) = \sin (\pi x)$ and we will extend the analysis of such products to other types of sequences $(x_k)_{k\ge 1}$. In particular we will consider two well-known types of uniformly distributed sequences, namely the van der Corput sequence $(x_k)_{k \ge 1}$ and the Kronecker sequence $(\{k \alpha \})_{k \ge 1}$ with irrational $\alpha \in [0,1]$. Furthermore, we will determine the typical behavior of

$$\begin{aligned} \prod ^{N}_{k=1} 2 \sin (\pi x_{k}), \end{aligned}$$

that is, the almost sure order of this product for “random” sequences $\left( x_{k}\right) _{k \ge 1}$ in a suitable probabilistic model.

Such sine-products and estimates for such products play an important role in many different fields of mathematics. We just mention a few of them: interpolation theory (see [18, 19]), partition theory (see [42, 48]), Padé approximation (see [33]), KAM theory and *q*-series (see [2, 15, 24, 26, 29]), analytic continuation of Dirichlet series (see [25, 45]), and many more.

All our results use methods from uniform distribution theory and discrepancy theory, so we will introduce some of the basic notions from these subjects. Let $x_1, \dots , x_N$ be numbers in [0, 1]. Their *star-discrepancy* is defined as

$$\begin{aligned} D_{N}^{*}=D_N^*(x_{1}, \ldots , x_{N}) = \sup _{a\in [0,1]} \left| \frac{A_{N}(a)}{N} -a \right| , \end{aligned}$$

where $A_{N} (a) := \# \left\{ 1 \le n \le N \ : \ x_n \in [0,a)\right\}$. An infinite sequence $(x_k)_{k \ge 1}$ in [0, 1] is called *uniformly distributed modulo one* (u.d. mod 1) if for all $a \in [0,1]$ we have

$$\begin{aligned} \lim _{N \rightarrow \infty } \frac{A_N(a)}{N} = a, \end{aligned}$$

or, equivalently,

$$\begin{aligned} \lim _{N \rightarrow \infty } D_N^* = 0. \end{aligned}$$

For more basic information on uniform distribution theory and discrepancy, we refer to [10, 28].

Now we come to our new results. First we will give general estimates for products $\prod ^{N}_{k=1} 2 \sin (\pi x_{k})$ in terms of the star-discrepancy $D_{N}^{*}$ of $(x_k)_{1 \le k \le N}$. A similar result in a weaker form was obtained by Hlawka [18] (see also [19]).

### Theorem 1

Let $\left( x_{k}\right) _{k \ge 1}$ be a sequence of real numbers from [0, 1] which is u.d. mod 1. Then for all sufficiently large *N* we have

3 $$\begin{aligned} \prod ^{N}_{k=1} 2 \sin (\pi x_{k}) \le \left( \frac{N}{\Delta _{N}}\right) ^{2 \Delta _{N}}, \end{aligned}$$

where $$\Delta _{N} := ND_{N}^{*}$$.

Concerning the quality of Theorem 1, consider the case when $(x_{k})_{k \ge 1}$ is a low-discrepancy sequence such as the van der Corput sequence (which is treated in Theorem 5 below). Then $\Delta _{N} = \mathcal {O}\left( \log N\right)$, and Theorem 1 gives

4 $$\begin{aligned} \prod ^{N}_{k=1} 2 \sin (\pi x_{k}) \le N^{\gamma \log N} \end{aligned}$$

for some $\gamma \in \mathbb {R}^+$ and all sufficiently large *N*. Stronger asymptotic bounds are provided by Theorem 5 below; thus, Theorem 1 does not provide a sharp upper bound in this case.

As another example, let $x_k=k/(N+1)$ for $k=1,2,\ldots ,N$. This point set has star-discrepancy $D_N^*=1/(N+1)$, and hence the general estimate (3) gives

5 $$\begin{aligned} \prod ^{N}_{k=1} 2 \sin \left( \pi \frac{k}{N+1}\right) \le (N+1)^2. \end{aligned}$$

To be precise we can obtain this estimate directly from Theorem 1 only for “infinitely many *N*” instead of “for arbitrary *N*”.

Theorem 1 is stated for sequences, hence the “sufficiently large *N*” may depend on the sequence. But we can apply the Theorem 1 to a sequence $(x_k)_{k \ge 1}$ which is designed such that for infinitely many *N* we have $x_k = k / (N + 1)$ for $k = 1, 2, \ldots , N$.

On the other hand, the product on the left-hand side of (5) is well known to be exactly $N+1$ (see also Lemma 3 below). Thus, the general estimate from Theorem 1 has an additional factor *N* in comparison with the correct order in this case, which is quite close to optimality.

As already mentioned above, Hlawka [18, 19] studied similar questions in connection with interpolation of analytic functions on the complex unit disc. There he considered products of the form

$$\begin{aligned} \omega _{N}(z)= \prod ^{N}_{k=1}(z-\xi _{k})^{2}, \end{aligned}$$

where $\xi _{k}$ are points on the unit circle. The main results in [18, 19] are lower and upper bounds of $|\omega _{N}(z)|$ in terms of the star-discrepancy $D_{N}^*$ of the sequence $(\arg \frac{1}{2\pi }\xi _{k}), k=1,\ldots , N.$ 1 It should also be mentioned that Wagner [45] proved the general lower bound

$$\begin{aligned} \sup _{| z|= 1}|\omega _{N}(z)|\ge (\log N)^{c} \end{aligned}$$

for infinitely *N*, where $c> 0$ is some explicitly given constant. This solved a problem stated by Erdős.

In the sequel we will give a second, essentially optimal theorem which estimates products $\prod ^{N}_{k=1} 2 \sin (\pi x_{k})$ in terms of the star-discrepancy of the sequence $(x_{k})_{k \ge 1}$. Let $\omega =\left\{ x_{1}, \ldots , x_{N}\right\}$ be numbers in [0, 1] and let $P_N(\omega )=\prod ^{N}_{k=1} 2 \sin (\pi x_k)$. Let $D_{N}^{*} (\omega )$ denote the star-discrepancy of $\omega$. Furthermore, let $d_N$ be a real number from the interval [1 / (2*N*), 1], which is the possible range of the star-discrepancy of *N*-element point sets. We are interested in

$$\begin{aligned} P_N^{(d_{N})} := \sup _{\omega } P_N(\omega )= \sup _{\omega } \prod ^{N}_{k=1}2 \sin (\pi x_k), \end{aligned}$$

where the supremum is taken over all $\omega$ with $D_{N}^{*} (\omega ) \le d_{N}$. We will show

### Theorem 2

Let $(d_N)_{N \ge 1}$ be an arbitrary sequence of reals of the form $d_N = \frac{M (N)}{N}$ with *M*(*N*) positive integers, and $\lim _{N \rightarrow \infty } d_N = 0$. Then we have:

- For all $\varepsilon > 0$ there exist $c(\varepsilon )$ and $N(\varepsilon )$ such that for all $N > N(\varepsilon )$ we have
  $$\begin{aligned} P_N^{(d_{N})} \le c(\varepsilon ) \frac{1}{N} \left( \left( \frac{\mathrm{e}}{\pi }+\varepsilon \right) \frac{1}{d_{N}}\right) ^{2N d_{N}}. \end{aligned}$$
- For all sufficiently large *N* we have
  $$\begin{aligned} P_N^{(d_{N})} \ge \frac{2 \pi ^2}{\mathrm{e}^6} \frac{1}{N} \left( \frac{\mathrm{e}}{\pi } \frac{1}{d_N}\right) ^{2 N d_N}. \end{aligned}$$

Let us now focus on products of the form

$$\begin{aligned} \prod ^{N}_{n=1} 2 \sin (\pi \{n \alpha \}) =\prod ^{N}_{n=1} \left| 2 \sin (\pi n \alpha )\right| , \end{aligned}$$

where $\alpha$ is a given irrational number, i.e., we consider the special case when $(x_n)_{n \ge 1}$ is the Kronecker sequence $(\{n \alpha \})_{n \ge 1}$. Such products play an essential role in many fields and are the best studied such Weyl products in the literature. See for example [7, 9, 16, 21, 25, 32, 39, 44]. Before discussing these products in detail, let us recall some historical facts. By Kronecker’s approximation theorem, the sequence $(n\alpha )_{n \ge 1}$ is everywhere dense modulo 1; i.e., the sequence of fractional parts $(\{n\alpha \})_{n \ge 1}$ is dense in [0, 1]. At the beginning of the 20th century various authors considered this sequence (and generalizations such as $(\{\alpha n^{d}\})_{n \ge 1}$, etc.) from different points of view; see for instance Bohl [5], Weyl [46] and Sierpińksi [40]. An important impetus came from celestial mechanics. It was Hermann Weyl in his seminal paper [47] who opened new and much more general features of this subject by introducing the concept of uniform distribution for arbitrary sequences $(x_{k})_{k\ge 1}$ in the unit interval (as well as in the unit cube $[0,1]^{s}$). This paper heavily influenced the development of uniform distribution theory, discrepancy theory and the theory of quasi-Monte Carlo integration throughout the last 100 years. For the early history of the subject we refer to Hlawka and Binder [20].

Numerical experiments suggest that for integers *N* with $q_{l} \le N < q_{l+1}$, where $\left( q_{l}\right) _{l \ge 0}$ is the sequence of best approximation denominators of $\alpha$,

6 $$\begin{aligned} \text {the product attains its maximal value for}~N = q_{l+1}-1. \end{aligned}$$

Moreover we conjecture that always

7 $$\begin{aligned} \limsup _{q \rightarrow \infty } \frac{1}{q} \prod ^{q-1}_{n=1} |2 \sin (\pi n \alpha ) | < \infty . \end{aligned}$$

**Fig. 1:**
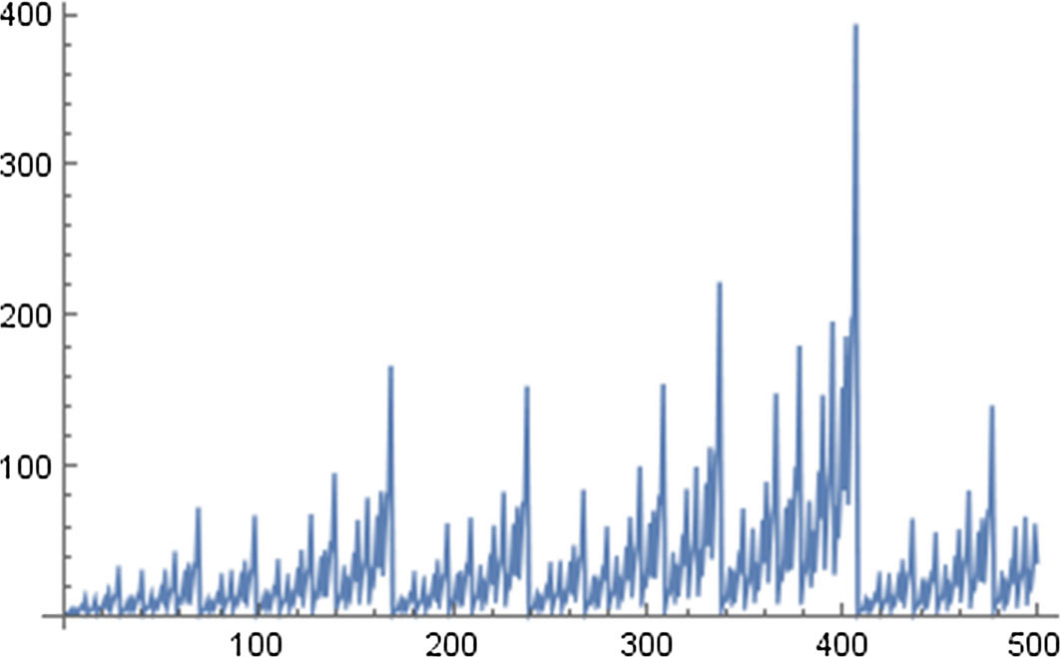
$$\prod ^{N}_{n=1} \left| 2 \sin (\pi n \alpha ) \right| $$ for $$N=1,\ldots , 500$$ and $$\alpha =\sqrt{2}$$

**Fig. 2:**
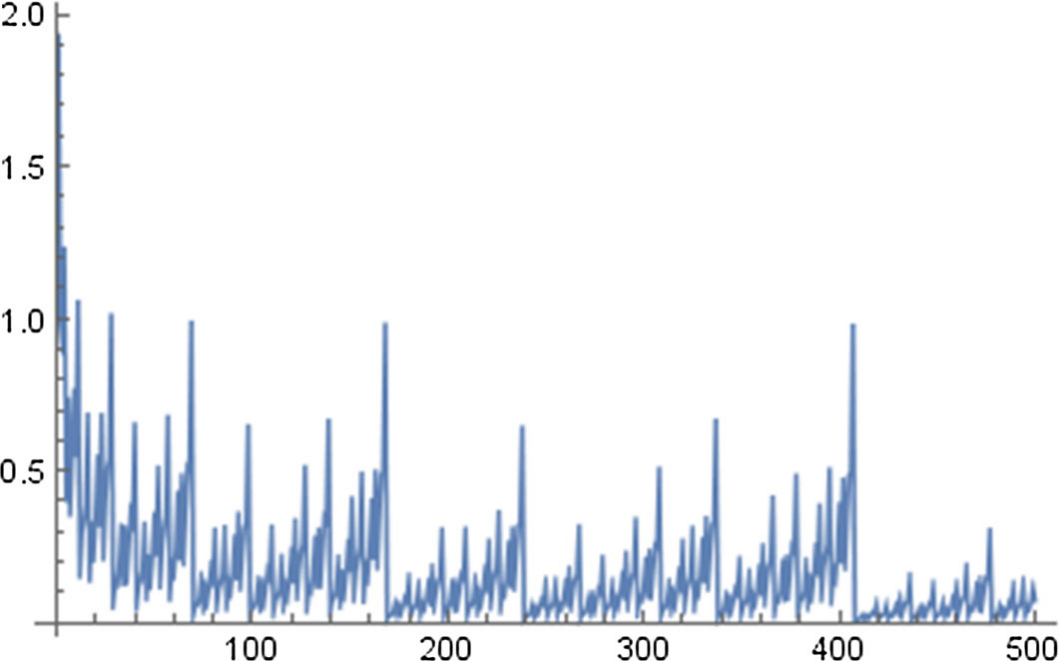
$$\tfrac{1}{N}\prod ^{N}_{n=1} \left| 2 \sin (\pi n \alpha ) \right| $$ for $$N=1,\ldots , 500$$ and $$\alpha =\sqrt{2}$$

Compare these considerations also with the conjectures stated in [32]. To illustrate these two assertions see Figs. 1 and 2, where for $\alpha = \sqrt{2}$ we plot $\prod ^{N}_{n=1} |2 \sin (\pi n \alpha )|$ for $N=1,\ldots , 500$ (Fig. 1) and the normalized version $\tfrac{1}{N} \prod ^{N}_{n=1} |2 \sin (\pi n \alpha ) |$ for $N=1, \ldots , 500$ (Fig. 2). Note that the first best approximation denominators of $\sqrt{2}$ are given by $1,2,5,12,29,70,169,408,\ldots$.

For the case $N= q-1$ for some best approximation denominator *q* the product $\prod ^{q-1}_{n=1} |2 \sin (\pi n \alpha )|$ already was considered in [9, 39], and in much more general form in [3] (see also [37]). In particular, it follows from the results given there that

8 $$\begin{aligned} \lim _{q \rightarrow \infty } \frac{1}{q}\log \prod ^{q-1}_{n=1} |2 \sin (\pi n \alpha )| = \lim _{q \rightarrow \infty } \frac{1}{q} \sum ^{q-1}_{n=1} \log |2 \sin (\pi n \alpha )| = 0, \end{aligned}$$

when *q* runs through the sequence of best approximation denominators. Indeed, we are neither able to prove assertion (6) nor assertion (7). Nevertheless we want to give a quantitative estimate for the case $N=q-1$, i.e., also a quantitative version of (8), before we will deal with the general case.

### Theorem 3

Let *q* be a best approximation denominator for $\alpha$. Then

$$\begin{aligned} 1 \le \prod ^{q-1}_{n=1} |2 \sin (\pi n \alpha )| \le \frac{q^{2}}{2}. \end{aligned}$$

Next we consider general $N\in \mathbb {N}$:

### Theorem 4

Let $\alpha := [0; a_1, a_2,a_3, \ldots ]$ be the continued fraction expansion of the irrational number $\alpha \in [0,1]$. Let $N \in \mathbb {N}$ be given, and denote its Ostrowski expansion by

$$\begin{aligned} N = b_{l}q_{l} + b_{l-1} q_{l-1} + \cdots + b_{1}q_{1} + b_{0} \end{aligned}$$

where $l=l(N)$ is the unique integer such that $q_l \le N < q_{l+1}$, where $b_{i} \in \{0,1,\ldots ,a_{i+1}\}$, and where $q_1,q_2,\ldots$ are the best approximation denominators for $\alpha$. Then we have

$$\begin{aligned} \prod ^{N}_{n=1} \left| 2 \sin (\pi n \alpha ) \right| \le \prod ^{l}_{i=0} 2^{b_{i}} q^{3}_{i}. \end{aligned}$$

### Corollary 1

For all *N* with $q_{l} \le N < q_{l+1}$ we have

$$\begin{aligned} \frac{1}{N} \sum ^{N}_{n=1} \log |2 \sin (\pi n \alpha )| \le (\log 2) \left( \frac{1}{q_{l}} + \frac{l}{2^{(l-3)/2}}\right) + 3 \, \frac{\log q_{l}}{q_{l}} \left( \frac{\log q_l}{\log \phi }+1 \right) , \end{aligned}$$

where $\phi =(1+\sqrt{5})/2$ and hence

$$\begin{aligned} \limsup _{N \rightarrow \infty } \frac{1}{N} \sum ^{N}_{n=1} \log |2 \sin (\pi n \alpha )| = 0 =\int _0^1 \log (2 \sin (\pi x)) \,\mathrm{d}x . \end{aligned}$$

The second part of Corollary 1 can also be obtained from [7, Lemma 4].

In the following we say that a real $\alpha$ is of type $t \ge 1$ if there is a constant $c > 0$ such that

$$\begin{aligned} \left| \alpha - \frac{p}{q}\right| > c \frac{1}{q^{1+t}} \end{aligned}$$

for all $p,q \in \mathbb {Z}$ with $\gcd (p,q)=1$.

The next result essentially improves a result given in [25]. There a bound on $\prod ^{N}_{n=1} \left| 2 \sin \left( \pi n \alpha \right) \right|$ for $\alpha$ of type *t* of the form $N^{c N^{1-1/t} \log N}$ instead of our much sharper bound $2^{C N^{1-1/t}}$ was given. Note that our result only holds for $t > 1$, so we cannot obtain the sharp result of Lubinsky [32] in the case of $\alpha$ with bounded continued fraction coefficients.

### Corollary 2

Assume that $$\alpha $$ is of type $$ t > 1$$. Then for some constant *C* and all *N* large enough $$\prod ^{N}_{n=1} \left| 2 \sin \left( \pi n \alpha \right) \right| \le 2^{C N^{1-1/t}}$$.

Now we will deal with $\prod ^{N}_{n=1} |2 \sin (\pi x_n)|$, where $(x_n)_{n \ge 1}$ is the van der Corput-sequence. The van der Corput sequence (in base 2) is defined as follows: for $n \in \mathbb {N}$ with binary expansion $n=a_0+a_1 2+a_2 2^3+\cdots$ with digits $a_0,a_1,a_2,\ldots \in \{0,1\}$ (of course the expansion is finite) the $n^{\mathrm{th}}$ element is given as

$$\begin{aligned} x_n=\frac{a_0}{2}+\frac{a_1}{2^2}+\frac{a_2}{2^3}+\cdots \end{aligned}$$

(see the recent survey [11] for detailed information about the van der Corput sequence). For this sequence, in contrast to the Kronecker sequence, we can give very precise results. We show:

### Theorem 5

Let $(x_{n})_{n \ge 1}$ be the van der Corput sequence in base 2. Then

$$\begin{aligned} \limsup _{N \rightarrow \infty } \frac{1}{N^{2}} \prod ^{N}_{n=1} |2 \sin (\pi x_{n})| = \frac{1}{2 \pi } \end{aligned}$$

and

$$\begin{aligned} \liminf _{N \rightarrow \infty } \prod ^{N}_{n=1} |2 \sin (\pi x_{n})| = \pi . \end{aligned}$$

Finally, we study probabilistic analogues of Weyl products, in order to be able to quantify the typical order of such products for “random” sequences and to have a basis for comparison for the results obtained for deterministic sequences in Theorems 3–5. We will consider two probabilistic models. First we study

$$\begin{aligned} \prod ^{N}_{k=1}2\sin (\pi X_{k}), \end{aligned}$$

where $(X_{k})_{k \ge 1}$ is a sequence of independent, identically distributed (i.i.d.) random variables in [0, 1]. The second probabilistic model are random subsequences $(n_{k}\alpha )_{k \ge 1}$ of the Kronecker sequences $(n\alpha )$, where the elements of $n_{k}$ are selected from $\mathbb {N}$ independently and with probability $\frac{1}{2}$ for each number. This model is frequently used in the theory of random series (see for example the monograph of Kahane [23]) and was introduced to the theory of uniform distribution by Petersen and McGregor [38] and later extensively studied by Tichy [43], Losert [30], and Losert and Tichy [31].

### Theorem 6

Let $(X_{k})_{k \ge 1}$ be a sequence of i.i.d. random variables having uniform distribution on [0, 1], and let

$$\begin{aligned} P_{N}= \prod ^{N}_{k=1}2\sin (\pi X_{k}). \end{aligned}$$

Then for all $\varepsilon > 0$ we have, almost surely,

$$\begin{aligned} P_N \le \exp \left( \left( \frac{\pi }{\sqrt{6}} + \varepsilon \right) \sqrt{N \log \log N}\right) \end{aligned}$$

for all sufficiently large *N*, and

$$\begin{aligned} P_N \ge \exp \left( \left( \frac{\pi }{\sqrt{6}}-\varepsilon \right) \sqrt{N \log \log N}\right) \end{aligned}$$

for infinitely many *N*.

### Theorem 7

Let $\alpha$ be an irrational number with bounded continued fraction coefficients. Let $(\xi _n)_{n \ge 1}=(\xi _n(\omega ))_{n \ge 1}$ be a sequence of i.i.d. $\{0,1\}$-valued random variables with mean 1 / 2, defined on some probability space $(\Omega ,\mathcal {A},\mathbb {P})$, which induce a random sequence $(n_k)_{k \ge 1}=(n_k(\omega ))_{k \ge 1}$ as the sequence of all numbers $\left\{ n \ge 1:~ \xi _n = 1 \right\}$, sorted in increasing order. Set

$$\begin{aligned} P_N = \prod ^{N}_{k=1}2\sin (\pi n_k \alpha ). \end{aligned}$$

Then for all $\varepsilon > 0$ we have, $\mathbb {P}$-almost surely,

$$\begin{aligned} P_N \le \exp \left( \left( \frac{\pi }{\sqrt{12}} + \varepsilon \right) \sqrt{N \log \log N}\right) \end{aligned}$$

for all sufficiently large *N*, and

$$\begin{aligned} P_N \ge \exp \left( \left( \frac{\pi }{\sqrt{12}}-\varepsilon \right) \sqrt{N \log \log N}\right) \end{aligned}$$

for infinitely many *N*

### Remark 1

The conclusion of Theorem 7 remains valid if $\alpha$ is only assumed to be of finite approximation type (see [28, Chapter 2, Section 3] for details on this notion).

### Remark 2

It is interesting to compare the conclusions of Theorems 6 (for purely random sequences) and 7 (for randomized subsequences of linear sequences) to the results in equations (1) and (2), which hold for lacunary trigonometric products. The results coincide almost exactly, except for the constants in the exponential term (which can be seen as the standard deviations in a related random system; see the proofs). The larger constant in the lacunary setting comes from an interference phenomenon, which appears frequently in the theory of lacunary functions systems (see for example Kac [22] and Maruyama [34]). On the other hand, the smaller constant in Theorem 7 represents a “loss of mass” phenomenon, which can be observed in the theory of slowly growing (randomized) trigonometric systems; it appears in a very similar form for example in Berkes [4] and Bobkov–Götze [6]. It is also interesting that the constant $\pi /\sqrt{6}$ in Theorem 1 is exactly the same as in results obtained by Fukuyama [13] for products $\prod |2 \sin (\pi n_k \alpha )|$ and $\prod |2 \cos (\pi n_k \alpha )|$ under the “super-lacunary” gap condition $n_{k+1}/n_k \rightarrow \infty$.

The outline of the remaining part of this paper is as follows. In Sect. 2 we will prove Theorems 1 and 2, which give estimates of Weyl products in terms of the discrepancy of the numbers $(x_k)_{1 \le k \le N}$. In Sect. 3 we prove the results for Kronecker sequences (Theorems 3 and 4), and in Sect. 4 the results for the van der Corput sequence (Theorem 5). Finally, in Sect. 5 we prove the results about probabilistic sequences (Theorems 6 and 7).

## Proofs of Theorems 1 and 2

### Proof of Theorem 1

The Koksma–Hlawka-inequality (see e.g. [28]) states that for any function $g:[0,1] \rightarrow \mathbb {R}$ of bounded variation *V*(*g*), any *N* and numbers $x_1, \dots , x_N \in [0,1]$ we have

$$\begin{aligned} \left| \int ^{1}_{0} g(x) \,\mathrm{d}x - \frac{1}{N} \sum ^{N}_{k=1} g\left( x_{k}\right) \right| \le V(g) \, D^{*}_{N} (x_1, \dots , x_N), \end{aligned}$$

where $D_{N}^{*}$ is the star-discrepancy of $x_{1}, \ldots , x_{N}$. Let $P_{N} := \prod ^{N}_{k=1} 2 \sin ( \pi x_{k})$ and

$$\begin{aligned} \Sigma _{N} := \log P_{N} = N \log 2 + \sum ^{N}_{k=1} \log \sin (\pi x_{k}). \end{aligned}$$

For $$0< \varepsilon < \frac{1}{2}$$ let

$$\begin{aligned} f_{\varepsilon } (x) := \left\{ \begin{array}{ll} \log \sin (\pi \varepsilon ) &{} \quad \text{ if }~\left\| x\right\| \le \varepsilon \\ \log \sin (\pi x) &{} \quad \text{ otherwise. }\\ \end{array} \right. \end{aligned}$$

Note, that $\int ^{1}_{0} \log \sin (\pi x) \,\mathrm{d}x = - \log 2$, hence

$$\begin{aligned} \int ^{1}_{0} f_{\varepsilon } (x) ~\,\mathrm{d}x&= 2 \varepsilon \log \sin (\pi \varepsilon ) + \int ^{1}_{0} \log \sin (\pi x) \,\mathrm{d}x - 2 \int ^{\varepsilon }_{0} \log \sin (\pi x) \,\mathrm{d}x \\&= 2 \varepsilon \log \sin (\pi \varepsilon ) - \log 2 - 2 \int ^{\varepsilon }_{0} \log \sin (\pi x) \,\mathrm{d}x. \end{aligned}$$

By partial integration we obtain

$$\begin{aligned} \int ^{\varepsilon }_{0} \log \sin (\pi x) \,\mathrm{d}x&= \varepsilon \log \sin (\pi \varepsilon ) - \int ^{\varepsilon }_{0} x \pi \cot (\pi x) \,\mathrm{d}x \\&= \varepsilon \log \sin (\pi \varepsilon ) - \varepsilon - \mathcal {O}(\varepsilon ^{3}) \end{aligned}$$

(with a positive $\mathcal {O}$-constant for $\varepsilon$ small enough). Furthermore, we have

$$\begin{aligned} V(f_{\varepsilon }) = \int _0^1 |f_{\varepsilon }'(x)| \,\mathrm{d}x = 2 \pi \int _{\varepsilon }^{1/2} \cot (\pi x) \,\mathrm{d}x = -2 \log \sin (\pi \varepsilon ). \end{aligned}$$

Altogether we have, using the Koksma-Hlawka inequality and since $\log \sin (\pi \varepsilon ) = \log (\pi \varepsilon ) - \frac{\pi ^{2} \varepsilon ^{2}}{6} - \mathcal {O}(\varepsilon ^{4}),$

$$\begin{aligned} \Sigma _{N}&\le N \log 2 + \sum ^{N}_{k=1} f_{\varepsilon } \left( x_{k}\right) \\&\le N \log 2 + N \int ^{1}_{0} f_{\varepsilon } (x) \,\mathrm{d}x + N D_{N}^{*} V(f_{\varepsilon })\\&= N\left( 2 \varepsilon \log \sin \pi \varepsilon - 2 \int ^{\varepsilon }_{0} \log \sin (\pi x) \,\mathrm{d}x \right) - 2 N D_{N}^{*} \log \sin (\pi \varepsilon ) \\&= 2N \int ^{\varepsilon }_{0} x \pi \cot (\pi x) \,\mathrm{d}x - 2ND_{N}^{*} \log \sin (\pi \varepsilon ) \\&= 2 N \varepsilon + N \mathcal {O}(\varepsilon ^{3}) + 2 N D_{N}^{*} \, (-\log (\pi \varepsilon ) + \mathcal {O} (\varepsilon ^{2})) \\&= 2 N \varepsilon - 2 N D_{N}^{*} \log \pi \varepsilon + N \mathcal {O}(\varepsilon ^{2}). \end{aligned}$$

Hence

$$\begin{aligned} P_{N}=\mathrm{e}^{\Sigma _N} \le \mathrm{e}^{2 N \varepsilon } \left( \frac{1}{\pi \varepsilon }\right) ^{2 N D_{N}^{*}} \mathrm{e}^{c \varepsilon ^{2} N} \end{aligned}$$

for some constant $c>0$. We choose $\varepsilon = D_{N}^{*}$ and obtain

$$\begin{aligned} P_{N} \le \left( c' \, \frac{N}{N D_{N}^{*}}\right) ^{2 ND_{N}^{*}} \end{aligned}$$

For some $c'>0$. Note that $c'$ can be chosen such that $c' < 1$ if $\varepsilon = D_{N}^{*} =o(1)$ for $N \rightarrow \infty$. $\square$

Next we come to the proof of Theorem 2. We will need several auxiliary lemmas, before proving the theorem.

### Lemma 1

For $N, M \in \mathbb {N}$ with $M \le \frac{N}{2}$ let $D:= \frac{M}{N}$. Consider the following point set $\widetilde{\omega }$: If *N* is even, the $\widetilde{\omega }$ is given by the points

$$\begin{aligned} \frac{M}{N}, \frac{M+1}{N}, \ldots , \frac{\frac{N}{2}-1}{N}, \frac{\frac{N}{2}+1}{N}, \frac{\frac{N}{2}+2}{N}, \ldots , \frac{N-M}{N} \end{aligned}$$

together with 2*M* times the point $\frac{1}{2}$.

If *N* is odd, the $\widetilde{\omega }$ is given by

$$\begin{aligned} \frac{M}{N}, \frac{M+1}{N}, \ldots , \frac{\frac{N-1}{2}}{N}, \frac{\frac{N+1}{2}}{N}, \frac{\frac{N+3}{2}}{N}, \ldots , \frac{N-M}{N} \end{aligned}$$

together with $2M-1$ times the point $\frac{1}{2}$.

(i) Then $\widetilde{\omega }$ has star-discrepancy
  $$\begin{aligned} D_{N}^{*} \left( \widetilde{\omega }\right) =D. \end{aligned}$$
(ii) If any of the points of $\widetilde{\omega }$ is moved nearer to $\frac{1}{2}$, then the star-discrepancy of the new point set is larger than D.

### Proof

**Fig. 3:**
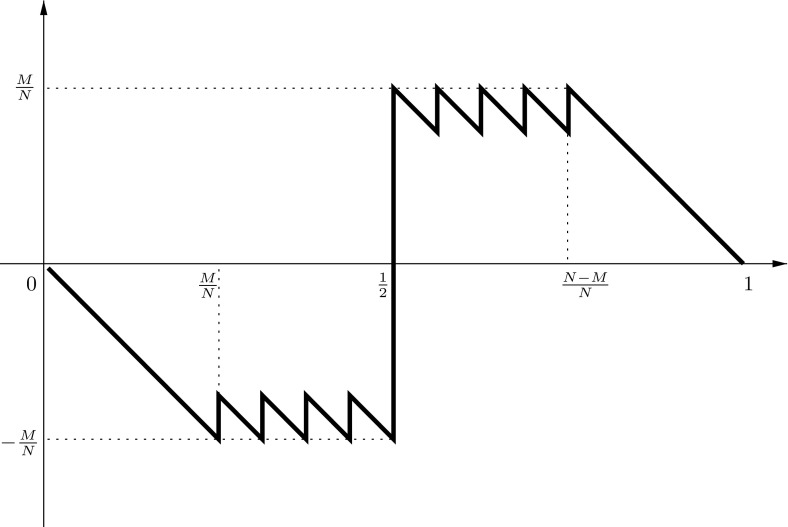
Discrepancy function $a \mapsto \frac{A_{N}(a)}{N} -a$ of $\widetilde{\omega }$

We give the proof for *N* even only (the proof for *N* odd runs quite analogously). The parts (i) and (ii) immediately follow from the form of the graph of the discrepancy function $a \rightarrow \frac{A_{N} (a)}{a} -a$ for $a \in \left[ 0,1\right]$ as it is plotted in Fig. 3.$\square$

### Lemma 2

For $\widetilde{\omega }$ as in Lemma 1 we have $P_N^{(d_{N})} = P_N(\widetilde{\omega })$.

### Proof

Let $\widetilde{x}_{1} \le \widetilde{x}_{2} \le \cdots \le \widetilde{x}_{N}$ denote the points of $\widetilde{\omega }$. Assume, there is another *N*-point set $\omega$ different from $\widetilde{\omega }$ with points $x_{1} \le x_{2} \le \cdots \le x_{N}$ such that $D_{N}^{*} (\omega ) \le d_{N}$ and $P_{N}(\omega ) > P_{N} \left( \widetilde{\omega }\right)$. Let $i \in \left\{ 1,\ldots , N\right\}$ be minimal such that $x_{i} \ne \widetilde{x}_{i}$, and assume that $\omega$ is chosen such that this $i=i(\omega )$ is maximal. If *i* is such that $\widetilde{x}_{i} < \frac{1}{2}$, then $x_{i} < \widetilde{x}_{i}$, otherwise (see Fig. 3) we had $D_{N}^{*}(\omega ) > d_{N}$.

By translating $x_{i}$ to $\widetilde{x}_{i}$ we obtain a new point-set $\widehat{\omega }$ with $D_{N}^{*} \left( \widehat{\omega }\right) \le d_{N}, P_{N} \left( \widehat{\omega }\right) > P_{N} \left( \omega \right)$, and $i\left( \widehat{\omega }\right) > i (\omega )$, a contradiction.

In the analogous way we can argue if *i* is such that $\widetilde{x}_{i} = \frac{1}{2}$, or such that $\widetilde{x}_{i} > \frac{1}{2}$. Hence, such an $\omega$ cannot exist.$\square$

### Lemma 3

For all $N\in \mathbb {N}$ and all $x\in [0,1]$ we have

(i) $$\prod ^{N-1}_{k=1} 2 \sin (\pi k/N) = N$$, and
(ii) $$\prod ^{N-1}_{k=0} 2 \sin (\pi (k+x)/N) = 2 \sin (\pi x).$$

### Proof

The proof of Equation (ii) is based on noting that $\mathrm{e}^{iaN}$ and $\mathrm{e}^{-iaN}$ are the zeros of $X^{2} - 2\cos (aN) X+1$. Then, the polynomial $X^{2N} - 2 \cos (aN) X^{N} + 1$ has 2*N* zeros and these are

$$\begin{aligned} \cos \left( a+\frac{2 \pi k}{N}\right) \pm i \sin \left( a + \frac{2 \pi k}{N}\right) , \quad \left( k = 0,1, \ldots , N-1\right) . \end{aligned}$$

Hence, we get

$$\begin{aligned} X^{2N} - 2 \cos \left( aN\right) X^{N} + 1 = \prod ^{N-1}_{k=0} \left( X^{2} - 2 \cos \left( a + \frac{2 \pi k}{N}\right) X + 1\right) . \end{aligned}$$

Taking $X = 1$ and $a=2b$, the last equation is written as

$$\begin{aligned} 2 \sin \left( bN\right) = \prod ^{N-1}_{k=0} 2 \sin \left( b + \frac{\pi k}{N}\right) . \end{aligned}$$

This is a standard formula that can be found in [14, Formula 1.392].

Putting $b = \frac{\pi x}{N}$, the proof of assertion (ii) is complete. Equation (i) follows immediately from Equation (ii) by noting that

$$\begin{aligned} \frac{\sin (bN)}{\sin (b)} = \prod ^{N-1}_{k=1} 2 \sin \left( b + \frac{\pi k}{N}\right) . \end{aligned}$$

Letting $b \rightarrow 0$ and using l’Hospital’s rule, we conclude that

$$\begin{aligned} \prod ^{N-1}_{k=1} 2 \sin \left( \frac{\pi k}{N}\right) = N. \end{aligned}$$

Another nice proof of Equation (i) can be found for example in [35]. $\square$

### Lemma 4

There is an $\varepsilon _{0} > 0$ such that for all $\varepsilon < \varepsilon _{0}$ we have

$$\begin{aligned} \varepsilon \log (\pi \varepsilon ) - \varepsilon - \varepsilon ^{2} \le \int ^{\varepsilon }_{0} \log \sin (\pi x) \,\mathrm{d}x \le \varepsilon \log (\pi \varepsilon ) - \varepsilon . \end{aligned}$$

### Proof

This follows immediately from the Taylor expansion

$$\begin{aligned} \int ^{\varepsilon }_{0} \log \sin (\pi x) \,\mathrm{d}x - \varepsilon \log (\pi \varepsilon ) = - \varepsilon - \frac{\pi ^{2}}{18} \varepsilon ^{3} + \mathcal {O}(\varepsilon ^{5}). \end{aligned}$$

$$\square $$

### Lemma 5

There is an $\varepsilon _{0} > 0$ such that for all $\varepsilon < \varepsilon _{0}$ we have

$$\begin{aligned} \log (\pi \varepsilon ) - \varepsilon \le \log \sin (\pi \varepsilon ) \le \log (\pi \varepsilon ). \end{aligned}$$

**Fig. 4:**
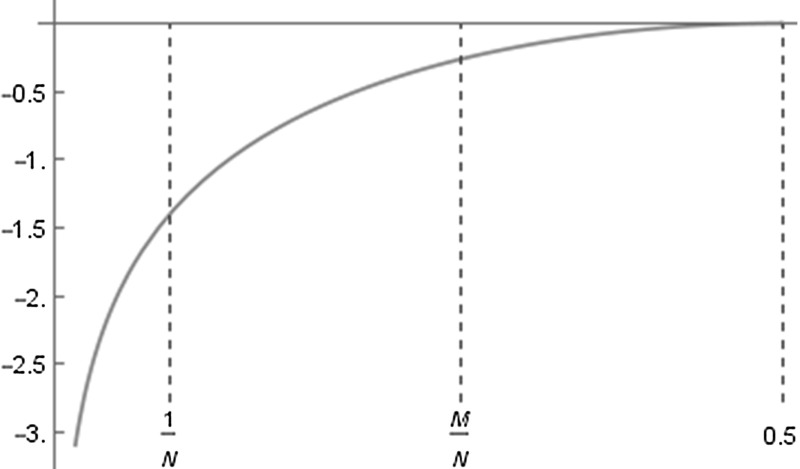
The function $$\log \sin (\pi x)$$

### Proof

This follows from

$$\begin{aligned} \log \sin (\pi x) - \log (\pi x) = - \frac{\pi ^{2}x^{2}}{6} + \mathcal {O}(x^{4}). \end{aligned}$$

$$\square $$

### Proof of Theorem 2

Let $N d_N = M$ with $M \ge 2$ (for $M=1$ the result is easily checked by following the considerations below) and $\widetilde{\omega }$ as in Lemmas 1 and 2. Note that $M=M(N)$ depends on *N*. We assume *M*(*N*) even. For *M*(*N*) odd the calculations are carried out quite analogously. We have, using also equation (i) of Lemma 3,

$$\begin{aligned} P_N(\widetilde{\omega })&= \left( \prod ^{N-1}_{k=1} 2 \sin \left( \pi \frac{k}{N}\right) \right) \ 2^{2 M-1} \ \left( \prod ^{M-1}_{k=1} 2 \sin \left( \pi \frac{k}{N}\right) \right) ^{-2}\\&= 2 N\left( \prod ^{M-1}_{k=1} \sin \left( \pi \frac{k}{N}\right) \right) ^{-2}. \end{aligned}$$

Note that the function $x \mapsto \log \sin (\pi x)$ is of the form as presented in Fig. 4. Hence for $M < \frac{N}{2}$ we have

$$\begin{aligned}&\log \sin \left( \frac{\pi }{N}\right) + N \int ^{\frac{M-1}{N}}_{\frac{1}{N}} \log \sin (\pi x) \,\mathrm{d}x\\&\quad \le \sum ^{M-1}_{k=1} \log \sin \left( \pi \frac{k}{N}\right) \\&\quad \le N \int ^{\frac{M-1}{N}}_{\frac{1}{N}} \log \sin (\pi x) \,\mathrm{d}x + \log \sin \left( \pi \frac{M-1}{N}\right) . \end{aligned}$$

By Lemma 4 for all *M* with $\frac{M}{N} < \varepsilon _{0}$ for the integral above we have

$$\begin{aligned}&N \int ^{\frac{M-1}{N}}_{\frac{1}{N}} \log \sin (\pi x) \,\mathrm{d}x\\&\quad \le \left( M-1\right) \log \left( \pi \frac{M-1}{N}\right) - (M-1) - \log \left( \frac{\pi }{N}\right) + 1 + \frac{1}{N}, \end{aligned}$$

and hence, using also Lemma 5,

$$\begin{aligned} \sum ^{M-1}_{k=1} \log \sin \left( \pi \frac{k}{N} \right)&\le (M-1) \log \left( \frac{\pi }{\mathrm{e}} \frac{M-1}{N}\right) - \log \pi +\log N + 1\\&\quad + \frac{1}{N} + \log (M-1) - \log N + \log \pi \\&\le (M-1) \log \left( \frac{\pi }{\mathrm{e}} \frac{M-1}{N}\right) + \log (M-1) + 2. \end{aligned}$$

This gives

$$\begin{aligned} \left( \prod ^{M-1}_{k=1} \sin \left( \pi \frac{k}{N}\right) \right) ^{2} = \mathrm{e}^{2 \sum ^{M-1}_{k=1} \log \sin (\pi k/N)} \le \mathrm{e}^{4} (M-1)^{2} \left( \frac{\pi }{\mathrm{e}} \frac{M-1}{N}\right) ^{2 (M-1)}, \end{aligned}$$

and consequently

$$\begin{aligned} P_N(\widetilde{\omega })&\ge 2N \frac{1}{\mathrm{e}^{4}} \frac{1}{(M-1)^2} \left( \frac{\mathrm{e}}{\pi } \frac{N}{M-1}\right) ^{2(M-1)} = \frac{2 \pi ^2}{\mathrm{e}^6} \frac{1}{N} \left( \frac{\mathrm{e}}{\pi } \frac{N}{M-1}\right) ^{2 M}\nonumber \\&\ge \frac{2 \pi ^2}{\mathrm{e}^6} \frac{1}{N} \left( \frac{\mathrm{e}}{\pi } \frac{1}{d_N}\right) ^{2 N d_N}. \end{aligned}$$

This proves assertion (b) of Theorem 2.

On the other hand we have

$$\begin{aligned}&N \int ^{\frac{M-1}{N}}_{\frac{1}{N}} \log \sin (\pi x) \,\mathrm{d}x\\&\qquad \ge (M-1) \log \left( \pi \frac{M-1}{N}\right) -(M-1) - \frac{(M-1)^{2}}{N} - \log \left( \frac{\pi }{N}\right) +1, \end{aligned}$$

and hence

$$\begin{aligned} \sum ^{M-1}_{k=1} \log \sin \left( \pi \frac{k}{N}\right)&\ge (M-1) \log \left( \frac{\pi }{\mathrm{e}} \frac{M-1}{N}\right) \\&\quad - \frac{(M-1)^{2}}{N} - \log \pi + \log N + 1 + \log \pi - \log N - \frac{1}{N}\\&= (M-1) \log \left( \frac{\pi }{\mathrm{e}} \frac{M-1}{N}\right) + 1-\frac{1}{N} - \frac{(M-1)^{2}}{N}. \end{aligned}$$

This gives

$$\begin{aligned} \left( \prod ^{M-1}_{k=1} \sin \left( \pi \frac{k}{N}\right) \right) ^{2} = \mathrm{e}^{2 \sum ^{M-1}_{k=1} \log \sin (\pi k/N)} \ge \frac{1}{\mathrm{e}^{\frac{2 (M-1)^{2}}{N}}} \left( \frac{\pi }{\mathrm{e}} \frac{M-1}{N}\right) ^{2(M-1)}, \end{aligned}$$

and consequently

9 $$\begin{aligned} P_N \left( \widetilde{\omega }\right) \le 2 N \mathrm{e}^{2 \frac{(M-1)^{2}}{N}} \left( \frac{\mathrm{e}}{\pi } \frac{N}{M-1}\right) ^{2 (M-1)}. \end{aligned}$$

It remains to show that for all $\varepsilon > 0$ there are $c(\varepsilon )$ and $N(\varepsilon )$ such that for all $N \ge N(\varepsilon )$ the right hand side of (9) is at most $c(\varepsilon ) \frac{1}{N} ((\tfrac{\mathrm{e}}{\pi } + \varepsilon ) \frac{N}{M})^{2 M}$.

To this end let $B(\varepsilon )$ be large enough such that for all $M > B(\varepsilon )$ we have $(M-1)^{1/M} \frac{M}{M-1} < 1 + \frac{\pi }{2 \mathrm{e}} \varepsilon$. Furthermore, let $N(\varepsilon )$ be large enough such that for all $N \ge N(\varepsilon )$ the value $\frac{M}{N} = d_{N}$ is so small such that

$$\begin{aligned} \mathrm{e}^{\frac{M-1}{N}} < \frac{1+\frac{\pi }{\mathrm{e}} \varepsilon }{1 + \frac{\pi }{2 \mathrm{e}} \varepsilon }. \end{aligned}$$

Then for all $M > B(\varepsilon )$ and all $N > N(\varepsilon )$ we have

$$\begin{aligned}&2 N \mathrm{e}^{2 \frac{\left( M-1\right) ^{2}}{N}} \left( \frac{\mathrm{e}}{\pi } \frac{N}{M-1}\right) ^{2 (M-1)}\\&\quad \le \frac{2 \pi ^2}{\mathrm{e}^2} \frac{\left( M-1\right) ^{2}}{N} \left( \frac{\mathrm{e}}{\pi } \, \mathrm{e}^{\frac{M-1}{N}} \frac{M}{M-1} \frac{N}{M} \right) ^{2 M}\\&\quad = \frac{2 \pi ^2}{\mathrm{e}^2 N} \left( \frac{\mathrm{e}}{\pi } \, \mathrm{e}^{\frac{M-1}{N}} \, (M-1)^{\frac{1}{M}} \, \frac{M}{M-1} \, \frac{N}{M}\right) ^{2 M}\\&\quad \le \frac{2 \pi ^2}{\mathrm{e}^2 N} \left( \left( \frac{\mathrm{e}}{\pi } + \varepsilon \right) \frac{N}{M}\right) ^{2 M}. \end{aligned}$$

If $M \le B(\varepsilon )$, then the penultimate expression can be estimated by

$$\begin{aligned}&\frac{2 \pi ^2}{\mathrm{e}^2N} \left( \frac{\mathrm{e}}{\pi } \, \mathrm{e}^{\frac{M-1}{N}} \, (M-1)^{\frac{1}{M}} \, \frac{M}{M-1} \, \frac{N}{M}\right) ^{2 M}\\&\quad \le \left( \max _{M\le B(\varepsilon )}\left( \frac{2 \pi ^2}{\mathrm{e}^2} \, \mathrm{e}^{2 M (M-1)} (M-1)^2 \left( \frac{M}{M-1}\right) ^{2 M}\right) \right) \frac{1}{N} \left( \frac{\mathrm{e}}{\pi } \frac{N}{M}\right) ^{2 M}\\&\quad = c(\varepsilon ) \frac{1}{N} \left( \frac{\mathrm{e}}{\pi } \frac{N}{M}\right) ^{2 M}, \end{aligned}$$

where

$$\begin{aligned} c(\varepsilon ):=\max _{M\le B(\varepsilon )}\left( \frac{2 \pi ^2}{\mathrm{e}^2} \, \mathrm{e}^{2 M \left( M-1\right) } \left( M-1\right) ^{2} \left( \frac{M}{M-1}\right) ^{2 M}\right) . \end{aligned}$$

This implies the desired result. $\square$

## Proofs of the results for Kronecker sequences

### Proof of Theorem 3

Let $\alpha = \frac{p}{q} + \theta$ with $0< \theta < \frac{1}{q \cdot q^{+}}$, where $q^{+}$ is the best approximation denominator following *q*. The case of negative $\theta$ can be handled quite analogously. There is exactly one of the points $\{k \alpha \}$ for $k=1, \dots , q-1$ in each interval $[\frac{m}{q}, \frac{m+1}{q})$ for $m=1, \ldots , q-1$. Note that the point in the interval $[\frac{q-1}{q}, 1)$ is the point $\left\{ q^{-} \alpha \right\}$, where $q^{-}$ is the best approximation denominator preceding *q*. We have

$$\begin{aligned} \left\{ q^{-} \alpha \right\} = \frac{q-1}{q} + q^{-} \theta \le \frac{q-1}{q} + \frac{q^{-}}{q \cdot q^{+}} < \frac{q-1}{q} + \frac{1}{2 q} = \frac{q- \frac{1}{2}}{q}. \end{aligned}$$

Hence, on the one hand (by equation (i) of Lemma 3),

$$\begin{aligned} \prod ^{q-1}_{n=1} |2 \sin ( \pi n \alpha )| \le \left( \prod ^{q-1}_{n=2} 2 \sin \left( \pi \frac{n}{q} \right) \right) \, 2 \sin \frac{\pi }{2} = \frac{2 q}{2 \sin (\pi /q)} \le \frac{q^2}{2}. \end{aligned}$$

On the other hand

$$\begin{aligned} \prod ^{q-1}_{n=1} |2 \sin (\pi n \alpha )|&\ge \left( \prod ^{q-1}_{n=1} 2 \sin \left( \pi \frac{n}{q}\right) \right) \ \frac{1}{2 \sin \left( \pi \tfrac{\lfloor q/2\rfloor }{q}\right) } \ \left| 2 \sin (\pi q^{-} \alpha )\right| \\&\ge q \, \sin \left( \pi \frac{q-1/2}{q}\right) = q \, \sin \frac{\pi }{2 q} \ge 1. \end{aligned}$$

$$\square $$

### Proof of Theorem 4

Let $N_{i} := b_{l}q_{l} + b_{l-1}q_{l-1} + \cdots + b_{i+1} q_{i+1}$ for $i=0, \ldots , l-1$ and $N_{l} := 0$. Then

$$\begin{aligned} \prod ^{N}_{n=1} \left| 2 \sin ( \pi n \alpha ) \right| = \prod ^{l}_{i=0} \prod ^{N_{i} + b_{i}q_{i}}_{n=N_{i}+1} \left| 2 \sin (\pi n \alpha ) \right| . \end{aligned}$$

We consider

$$\begin{aligned} \Pi _{i} := \prod ^{N_{i}+b_{i}q_{i}}_{n=N_{i}+1} \left| 2 \sin (\pi n \alpha ) \right| . \end{aligned}$$

Let $\alpha := \frac{p_{i}}{q_{i}} + \theta _{i}$ with, say, $\frac{1}{2 q_{i} q_{i+1}}< \theta _{i} < \frac{1}{q_{i} q_{i+1}}$. (The case of negative $\theta _{i}$ is handled quite analogously.)

Let $n=N_{i} + dq_{i} + k$ for some $0 \le d < b_{i}$ and $1 \le k \le q_{i}$, then, with $\kappa := \kappa _{i} := \{N_{i} \alpha \} \pmod {\frac{1}{q_{i}}}$ and $\tilde{\theta }_i:=q_i \theta _i$ we have

10 $$\begin{aligned} \left\{ n \alpha \right\} = \left\{ N_{i} \alpha + k \frac{p_{i}}{q_{i}} + (dq_{i}+k) \theta _{i} \right\} = \left\{ \kappa + \frac{l(k)}{q_{i}} + d \tilde{\theta }_i +k \theta _{i}\right\} \end{aligned}$$

for some $l(k)\in \{0,1,\ldots , q_i -1\}$. Since $0< k \theta _{i} + dq_{i} \theta _{i} \le \frac{a_{i+1}q_{i}}{q_{i+1}q_{i}} < \frac{1}{q_{i}}$, for given *d* there is always exactly one point $\left\{ n \alpha \right\}$ in the interval $[\kappa + \frac{l}{q_{i}} , \kappa + \frac{l+1}{q_{i}}) =: I_{l}$ for each $l=0,\ldots ,q_{i}-1$ (the interval taken modulo one).

We replace now the points $\left\{ n \alpha \right\}$ by new points, namely:

- if $\left\{ n \alpha \right\} \in I_{l}$ with $\kappa + \frac{l}{q_{i}} \ge \frac{1}{2}$ then in the representation (10) of $\left\{ n \alpha \right\}$ we replace $k \theta _{i}$ by 0, unless $l=q_{i}-1$.
- if $\left\{ n \alpha \right\} \in I_{l}$ with $\kappa + \frac{l+1}{q_{i}} < \frac{1}{2}$ then in the representation (10) of $\left\{ n \alpha \right\}$ we replace $k \theta _{i}$ by $\tilde{\theta }_{i}.$
- if $\left\{ n \alpha \right\} \in I_{l_{0}}$, where $l_{0}$ is such that $\kappa + \frac{l_{0}}{q_{i}} < \frac{1}{2} \le \kappa + \frac{l_{0}+1}{q_{i}}$ then
  - for the *d* such that $\kappa + \frac{l_{0}}{q_{i}} + d \tilde{\theta }_{i} \ge \frac{1}{2}$ in the representation (10) of $\left\{ n \alpha \right\}$ we replace $k \theta _{i}$ by 0,
  - for the *d* such that $\kappa + \frac{l_{0}}{q_{i}} + (d+1) \tilde{\theta }_{i} < \frac{1}{2}$ in the representation (10) of $\left\{ n \alpha \right\}$ we replace $k \theta _{i}$ by $\tilde{\theta }_{i}$,
  - for the single $d_{0}$ such that $\kappa + \frac{l_{0}}{q_{i}} + d_{0} \tilde{\theta }_{i} < \frac{1}{2} \le \kappa + \frac{l_{0}}{q_{i}} + \left( d_{0}+1\right) \tilde{\theta }_i$ we replace $\left\{ n \alpha \right\}$ by $\frac{1}{2}$.
- if $\left\{ n \alpha \right\} \in I_{l}$ with $l=q_{i}-1$, then
  - for the *h* such that $\kappa + \frac{q_{i}-1}{q_{i}} + h \tilde{\theta }_{i} \ge 1$ in the representation (10) of $\left\{ n\alpha \right\}$ we replace $k \theta _{i}$ by $\tilde{\theta }_{i}$,
  - for the *h* such that $\kappa + \frac{q_{i}-1}{q_{i}} + (h+1) \tilde{\theta }_{i} \le 1$ in the representation (10) of $\left\{ n \alpha \right\}$ we replace $k \theta _{i}$ by 0,
  - for the single $h_{0}$ such that $\kappa + \frac{q_{i}-1}{q_{i}} + h_{0} \tilde{\theta }_{i}< 1 < \kappa + \frac{q_{i}-1}{q_{i}} + \left( h_{0}+1\right) \tilde{\theta }_{i}$ we replace in the representation (10) of $\left\{ n \alpha \right\}$ the $k \theta _{i}$ by 0 if $g(\kappa + \frac{q_{i}-1}{q_{i}} + h_{0} \tilde{\theta }_{i}) \ge g(\kappa + \frac{q_{i}-1}{q} + (h_{0}+1) \tilde{\theta }_{i})$ and by $\tilde{\theta }_{i}$ otherwise, where here and in the following we use the notation $g(x) := \left| 2 \sin \pi x \right|$. Let the second be the case, the other case is handled quite analogously.

Using the new points instead of the $\left\{ n \alpha \right\}$ by construction we obtain an upper bound $\widetilde{\Pi }_{i}$ for $\Pi _{i}$. Then

$$\begin{aligned} \widetilde{\Pi }_{i}&= g(\kappa + \tilde{\theta }_{i} ) g(\kappa + 2 \tilde{\theta }_{i}) \cdots g(\kappa +b_{i} \tilde{\theta }_{i})\\&\quad \times g\left( \kappa + \tfrac{1}{q_{i}}+ \tilde{\theta }_{i}\right) g\left( \kappa +\tfrac{1}{q_{i}} + 2 \tilde{\theta }_{i}\right) \cdots g\left( \kappa + \tfrac{1}{q_{i}}+b_{i}\tilde{\theta }_{i}\right) \\&\quad \vdots \\&\quad \times g\left( \kappa + \tfrac{l_{0}-1}{q_{i}} + \tilde{\theta }_{i}\right) g\left( \kappa + \tfrac{l_{0}-1}{q_{i}} + 2 \tilde{\theta }_{i}\right) \cdots g\left( \kappa + \tfrac{l_{0}-1}{q_{i}} + b_{i} \tilde{\theta }_{i}\right) \\&\quad \times g\left( \kappa +\tfrac{l_{0}}{q_{i}} + \tilde{\theta }_{i}\right) \cdots g\left( \kappa + \tfrac{l_{0}}{q_{i}} + d_{0} \tilde{\theta }_{i}\right) g(\tfrac{1}{2}) \\&\quad \times g\left( \kappa + \tfrac{l_{0}}{q_{i}} + (d_{0}+1\right) \tilde{\theta }_{i}) \cdots g\left( \kappa + \tfrac{l_{0}}{q_{i}} + (b_{i}-1)\tilde{\theta }_{i}\right) \\&\quad \times g\left( \kappa + \tfrac{l_{0}+1}{q_{i}}\right) g\left( \kappa + \tfrac{l_{0}+1}{q_{i}} + \tilde{\theta }_{i}\right) \cdots g\left( \kappa + \tfrac{l_{0}+1}{q_{i}} + (b_{i}-1)\tilde{\theta }_{i}\right) \\&\quad \vdots \\&\quad \times g\left( \kappa + \tfrac{q_{i}-2}{q_{i}}\right) g\left( \kappa + \tfrac{q_{i}-2}{q_{i}} + \tilde{\theta }_{i}\right) \cdots g\left( \kappa + \tfrac{q_{i}-2}{q_{i}} + (b_{i}-1)\tilde{\theta }_{i}\right) \\&\quad \times g\left( \kappa + \tfrac{q_{i}-1}{q_{i}}\right) \cdots g\left( \kappa + \tfrac{q_{i}-1}{q_{i}} + (h_{0}-1) \tilde{\theta }_{i}\right) g\left( \kappa + \tfrac{q_{i}-1}{q_{i}} + (h_{0} + 1) \tilde{\theta }_{i}\right) \\&\quad \times g\left( \kappa + \tfrac{q_{i}-1}{q_{i}} + (h_{0}+2\right) \tilde{\theta }_{i}) \cdots g\left( \kappa + \tfrac{q_{i}-1}{q_{i}} + b_{i}\tilde{\theta }_{i}\right) . \end{aligned}$$

Hence

$$\begin{aligned} \widetilde{\Pi }_{i}&= \left( \prod ^{b_{i}-1}_{d=1} \prod ^{q_{i}-1}_{l=0} g \left( \kappa + \frac{l}{q_{i}} + d \tilde{\theta }_{i}\right) \right) \frac{g(\frac{1}{2})}{g(\kappa + \frac{q_{i}-1}{q_{i}} + h_{0} \tilde{\theta }_{i})} \\&\quad \times \left( \prod ^{l_{0}-1}_{l=0} g \left( \kappa + \frac{l}{q_{i}} + b_{i}\tilde{\theta }_{i}\right) \right) \prod ^{q_{i}-1}_{l=l_{0}+1} g \left( \kappa + \frac{l}{q_{i}}\right) . \end{aligned}$$

By equation (ii) of Lemma 3 we have

$$\begin{aligned} \prod ^{q_{i}-1}_{l=0} g \left( \kappa + \frac{l}{q_{i}} + d \tilde{\theta }_{i} \right) = 2 |\sin (\pi q_{i} (\kappa + d \tilde{\theta }_{i}))| \le 2 \end{aligned}$$

and hence

$$\begin{aligned} \prod ^{b_{i}-1}_{d=1} \prod ^{q_{i}-1}_{l=0} g \left( \kappa + \frac{l}{q_{i}} + d \tilde{\theta }_{i}\right) \le 2^{b_i-1} |\sin (\pi q_{i} (\kappa + h_{0} \tilde{\theta }_{i}))|. \end{aligned}$$

Note that $b_{i} \tilde{\theta }_i< \frac{a_{i+1}}{q_{i+1}} < \frac{1}{q_{i}}$ and therefore also $\kappa + d \tilde{\theta }_{i} < \frac{2}{q_{i}}$ always. Hence

$$\begin{aligned}&\left( \prod ^{l_{0}-1}_{l=0} g \left( \kappa + \frac{l}{q_{i}} + b_{i}\tilde{\theta }_{i}\right) \right) \prod ^{q_{i}-1}_{l=l_{0}+1} g \left( \kappa + \frac{l}{q_{i}}\right) \\&\quad \le g \left( \frac{2}{q_{i}}\right) g \left( \frac{3}{q_{i}}\right) \cdots g \left( \frac{\lfloor q_{i}/2\rfloor }{q_{i}}\right) g \left( \frac{1}{2}\right) ^{2} g \left( \frac{\lfloor q_{i}/2\rfloor +1}{q_{i}}\right) \cdots g \left( \frac{q_{i}-1}{q_{i}}\right) \\&\quad = \left( \prod ^{q_{i}-1}_{l=1} 2 \sin \left( \pi \frac{l}{q_{i}}\right) \right) \frac{4}{\sin (\pi /q_{i})}= \frac{4q_{i}}{\sin ( \pi /q_{i})} \le 2 q_{i}^{2}. \end{aligned}$$

Hence

$$\begin{aligned} \widetilde{\Pi }_{i} \le 2^{b_{i}-1} \, \frac{2 \left| \sin \left( \pi q_{i} \left( \kappa + h_{0} \tilde{\theta }_{i}\right) \right) \right| }{2 \left| \sin \left( \pi \left( \kappa + \tfrac{q_{i}-1}{q_{i}} + h_{0}\tilde{\theta }_{i}\right) \right) \right| } \, 2 q_{i}^{2}. \end{aligned}$$

We have

$$\begin{aligned} \frac{\left| \sin \left( \pi q_{i} \left( \kappa + h_{0} \tilde{\theta }_{i}\right) \right) \right| }{\left| \sin \left( \pi \left( \kappa + \tfrac{q_{i}-1}{q_{i}} + h_{0}\tilde{\theta }_{i}\right) \right) \right| }=\frac{\left| \sin \left( \pi q_{i} \left( \kappa +\tfrac{q_i -1}{q_i}+ h_{0} \tilde{\theta }_{i}\right) \right) \right| }{\left| \sin \left( \pi \left( \kappa + \tfrac{q_{i}-1}{q_{i}} + h_{0}\tilde{\theta }_{i}\right) \right) \right| } \le q_i, \end{aligned}$$

since $|\sin (nx)/\sin x| \le n$ for $n \in \mathbb {N}$. Hence

$$\begin{aligned} \widetilde{\Pi }_{i} \le 2^{b_{i}} q_{i}^{3} \end{aligned}$$

and therefore

$$\begin{aligned} \prod ^{N}_{n=1} |2 \sin (\pi n \alpha )| \le \prod ^{l}_{i=0} 2^{b_{i}} q_{i}^{3}, \end{aligned}$$

as desired. $$\square $$

### Proof of Corollary 1

By Theorem 4 we have

$$\begin{aligned} \frac{1}{N} \sum ^{N}_{n=1} \log |2 \sin (\pi n \alpha )|&\le (\log 2) \frac{b_{0} + \cdots + b_{l}}{b_{0} q_{0} + \cdots + b_{l} q_{l}} +3 \frac{\log q_{1} + \cdots + \log q_{l}}{b_{0} + b_{1}q_{1} + \cdots + b_{l}q_{l}} \\&\le (\log 2) \left( \frac{1}{q_{l}} + \frac{l \max _{0 \le i < l}b_{i}}{q_{l}}\right) + 3 \, \frac{l \log q_{l}}{q_{l}}. \end{aligned}$$

We have

$$\begin{aligned} q_{l} \ge a_{l}q_{l-1} + q_{l-2} \ge a_{l}a_{l-1}q_{l-2} + a_{l} q_{l-3} + q_{l-2} \ge \left( a_{l} a_{l-1} +1\right) q_{l-2}. \end{aligned}$$

By iteration we obtain

$$\begin{aligned} q_{l} \ge \left( a_{l} a_{l-1} +1\right) \left( a_{l-2} a_{l-3}+1\right) \cdots \left( a_{2} a_{1} +1\right) \ge 2^{\frac{l}{2}-1} \underset{1 \le i \le l}{\max }~a_{i} \end{aligned}$$

if *l* is even and

$$\begin{aligned} q_{l} \ge \left( a_{l} a_{l-1} +1\right) \left( a_{l-2}a_{l-3}+1\right) \cdots \left( a_{3} a_{2} +1\right) q_{1} \ge 2^{\frac{l-3}{2}} \underset{1 \le i \le l}{\max }~a_{i} \end{aligned}$$

if *l* is odd. With these estimates we get

$$\begin{aligned} \frac{1}{N} \sum ^{N}_{n=1} \log |2 \sin (\pi n \alpha )| \le (\log 2) \left( \frac{1}{q_{l}} + \frac{l}{2^{(l-3)/2}}\right) + 3 \, \frac{l \log q_{l}}{q_{l}}. \end{aligned}$$

Note that $q_l \ge \phi ^{l-1}$ and hence $l\le \frac{\log q_l}{\log \phi }+1$, where $\phi =(1+\sqrt{5})/2$.

Hence

$$\begin{aligned} \frac{1}{N} \sum ^{N}_{n=1} \log |2 \sin (\pi n \alpha )| \le (\log 2) \left( \frac{1}{q_{l}} + \frac{l}{2^{(l-3)/2}}\right) + 3 \, \frac{\log q_{l}}{q_{l}} \left( \frac{\log q_l}{\log \phi }+1 \right) . \end{aligned}$$

$$\square $$

### Proof of Corollary 2

Since $\alpha$ is of type $t >1$ we have

$$\begin{aligned} \frac{c}{q_{i}^{1+t}}< \left| \alpha - \frac{p_{i}}{q_{i}}\right| < \frac{1}{a_{i+1}q_{i}^{2}} \end{aligned}$$

and hence $b_{i} \le a_{i+1} < q_{i}^{t-1}/c$. Especially we have the following: Let $b_{l} := q_{l}^{\gamma }$, then, because of

$$\begin{aligned} q_{l}^{\gamma +1} = b_{l} q_{l} \le N < \left( b_{l}+1\right) q_{l} \le 2 q_{l}^{\gamma +1}, \end{aligned}$$

we have

$$\begin{aligned} b_{l} = q_{l}^{\gamma } \le N^{\frac{\gamma }{\gamma +1}} \le c_{1} N^{1-1/t}. \end{aligned}$$

Hence the bound from Theorem 4 can be estimated by

$$\begin{aligned} \prod ^{l}_{i=0} 2^{b_{i}} q_{i}^{3}\le & {} 2^{b_{l}} \left( \prod ^{l}_{i=0} q_{i}^{3} \right) \prod ^{l-1}_{i=0} 2^{b_{i}}\\\le & {} 2^{c_{1} N^{1-1/t}} N^{3 \left( l+1\right) } \prod ^{l-1}_{i=0} 2^{c_{1} N^{\left( 1-1/t\right) \left( 1/t\right) ^{i}}} \\\le & {} 2^{c_{2} N^{1-1/t}} N^{c_{3} \log N} \le 2^{C N^{1-1/t}} \end{aligned}$$

for *N* large enough. $\square$

## Proof of the result on the van der Corput sequence

Let

$$\begin{aligned} P_N:= \prod ^{N}_{k=1} 2 \sin (\pi x_k)~\text{ and }~f(k) := 2 \sin (\pi x_k), \end{aligned}$$

where $x_k$ is the $k^{\mathrm{th}}$ element of the van der Corput sequence.

### Lemma 6

Let (in dyadic representation)

$$\begin{aligned} n:= a_{s} a_{s-1} \ldots a_{k+1}\underbrace{ 0 1 1 \ldots 1 1}_{a_ka_{k-1}\ldots a_{l+1}} \underbrace{0 1 1 \ldots 1}_{a_l a_{l-1} \ldots a_0} \end{aligned}$$

and

$$\begin{aligned} \overline{n} := a_{s} a_{s-1} \ldots a_{k+1} 111 \ldots 11011 \ldots 1. \end{aligned}$$

Then $$P_{\overline{n}} > 2 P_{n}$$.

### Proof

We have

$$\begin{aligned} P_{\overline{n}}=P_n \, \frac{f(n+1) \cdots f(n+2^{l}) f(n+2^{l}+1)\cdots f(n+2^{l}+2^{k})}{f(n+2^{k}+1)\cdots f(n+2^{k}+2^{l})}. \end{aligned}$$

Since $$\{x_{n+1}, \ldots , x_{n+2^{l}}\} = \{\xi , \xi + \frac{1}{2^{l}}, \ldots , \xi + \frac{2^{l}-1}{2^{l}}\}$$ with

$$\begin{aligned} \xi =\frac{1}{2^{l+1}} + \cdots + \frac{1}{2^{k}} + \frac{a_{k+1}}{2^{k+2}} + \cdots + \frac{a_{s}}{2^{s+1}}, \end{aligned}$$

we obtain from equation (ii) of Lemma 3

$$\begin{aligned} f(n+1) \cdots f(n+2^{l}) = 2 \sin ( \pi 2^l \xi ). \end{aligned}$$

Furthermore, $$\{x_{n+2^{l}+1}, \ldots , x_{n+2^{l}+2^{k}}\}= \{y, y+\frac{1}{2^{k}}, \ldots , y + \frac{2^{k}-1}{2^{k}}\}$$ with

$$\begin{aligned} y=\frac{1}{2^{k+1}} + \frac{a_{k+1}}{2^{k+2}} + \cdots + \frac{a_{s}}{2^{s+1}} \end{aligned}$$

and hence, again by equation (ii) of Lemma 3,

$$\begin{aligned} f\left( n+2^{l}+1\right) \cdots f\left( n+2^{l}+2^{k}\right) = 2 \sin \left( \pi 2^k y\right) . \end{aligned}$$

Note that $$\frac{1}{2^{k+1}}< y < \frac{1}{2^{k}}$$.

In the same way we have $\{x_{n+2^{k}+1}, \ldots , x_{n+2^{k}+2^{l}}\} = \{\tau , \tau + \frac{1}{2^{l}}, \ldots , \tau + \frac{2^{l}-1}{2^{l}}\}$ with

$$\begin{aligned} \tau = \frac{1}{2^{l+1}} + \cdots + \frac{1}{2^{k+1}} + \frac{a_{k+1}}{2^{k+2}} + \cdots + \frac{a_{s}}{2^{s+1}} \end{aligned}$$

and hence by equation (ii) of Lemma 3

$$\begin{aligned} f(n + 2^{k}+1) \cdots f(n+2^{k}+2^{l}) = 2 \sin ( \pi 2^l \tau ). \end{aligned}$$

So

$$\begin{aligned} P_{\overline{n}}=P_n \frac{2 \sin (\pi 2^l \xi ) \sin (\pi 2^k y)}{\sin (\pi 2^l \tau )}. \end{aligned}$$

We have to show that

$$\begin{aligned} \Gamma := \frac{2 \sin (\pi 2^l \xi ) \sin (\pi 2^k y)}{\sin (\pi 2^l \tau )} > 2. \end{aligned}$$

Since $\tau = y + \frac{1}{2^{l}}-\frac{1}{2^{k}}$ and $\xi = y + \frac{1}{2^{l}}-\frac{1}{2^{k}}-\frac{1}{2^{k+1}}$ it follows that

$$\begin{aligned} \Gamma = \frac{2\sin \left( \pi \left( 2^{l} y+1-\frac{1}{2^{k-l}} - \frac{1}{2^{k+1-l}}\right) \right) \sin (\pi 2^{k}y)}{\sin \left( \pi \left( 2^{l} y+1-\frac{1}{2^{k-l}}\right) \right) }. \end{aligned}$$

Let $k-l =: m$ and $2^{l} y =: \eta$. Then we have $\frac{1}{2^{m+1}}<\eta <\frac{1}{2^{m}}$ and

$$\begin{aligned} \Gamma = \frac{2 \sin \left( \pi \left( \eta +1-\frac{1}{2^{m}} - \frac{1}{2^{m+1}}\right) \right) \sin (\pi 2^{m} \eta )}{\sin \left( \pi \left( \eta +1-\frac{1}{2^{m}}\right) \right) }. \end{aligned}$$

Let $z:= \frac{1}{2^{m}}-\eta$. Then we have $0< z < \frac{1}{2^{m+1}}$ and

$$\begin{aligned} \Gamma&= \frac{2 \sin \left( \pi \left( 1-z-\frac{1}{2^{m+1}}\right) \right) \sin (\pi (1-2^{m}z))}{\sin (\pi (1-z))} \\&=\frac{2 \sin \left( \pi \left( z+\frac{1}{2^{m+1}}\right) \right) \sin (\pi 2^m z)}{\sin (\pi z)} \\&> \frac{2 \sin \left( \pi \frac{1}{2^{m+1}}\right) \sin \left( \pi 2^{m} \frac{1}{2^{m+1}}\right) }{\sin \left( \pi \frac{1}{2^{m+1}}\right) } \\&= 2. \end{aligned}$$

Here we used that $\sin (\pi (z + \frac{1}{2^{m+1}}))$ for $0< z< \frac{1}{2^{m+1}}$ is minimal for $z \rightarrow 0$ and $\frac{\sin (\pi 2^{m} z)}{\sin ( \pi z)}$ for $0< z < \frac{1}{2^{m+1}}$ is minimal for $z\rightarrow \frac{1}{2^{m+1}}$. $\square$

### Lemma 7

We have:

(i) $$\begin{aligned} \hbox {Let}\quad n= & {} \overset{s}{\overset{\downarrow }{1}}111 \ldots 111\overset{k+1}{\overset{\downarrow }{0}}111 \ldots 1110 \\ \hbox {and} \quad \overline{n}= & {} 1111 \ldots 1111011 \ldots 1110 \\ \hbox {then} \, P_{\overline{n}}\ge & {} P_n. \end{aligned}$$
(ii) $$\begin{aligned} \hbox {Let}\quad n= & {} 1\overset{s-1}{\overset{\downarrow }{0}}11 \ldots 111\overset{k+1}{\overset{\downarrow }{0}}111 \ldots 1110 \\ \hbox {and}\quad \overline{n}= & {} 1011\ldots 1111011 \ldots 1110 \\ \hbox {then}\, P_{\overline{n}}\ge & {} P_n. \end{aligned}$$
(iii) $$\begin{aligned} \hbox {Let} \quad n= & {} 1111\ldots 111\overset{k+1}{\overset{\downarrow }{0}}111 \ldots 1111 \\ \hbox {and}\quad \overline{n}= & {} 1111\ldots 1111011\ldots 1111 \\ \hbox {then}\, P_{\overline{n}}\ge & {} P_n. \end{aligned}$$
(iv) $$\begin{aligned} \hbox {Let}\quad n= & {} 1011 \ldots 1110111 \ldots 1111 \\ \hbox {and}\quad \overline{n}= & {} 1011\ldots 1111011\ldots 1111 \\ \hbox {then}\, P_{\overline{n}}\ge & {} P_n. \end{aligned}$$

### Proof

We only prove (ii), which is the most elaborate part of the lemma. The other assertions can be handled in the same way but even simpler. In (ii) we have

$$\begin{aligned} P_{\overline{n}}&= P_n f(10111\ldots 110111 \ldots 111) \prod ^{2^{k}-2}_{i=0} f(1011\ldots 100\ldots 0+i) \\&=P_n 2 \sin \left( \pi \left( 1-\frac{1}{2^{k+2}}- \frac{3}{2^{s+1}}\right) \right) \frac{\sin (\pi x)}{\sin (\pi \frac{1-x}{2^{k}})} \end{aligned}$$

with $$x=2^k(\frac{1}{2^{k+1}} - \frac{3}{2^{s+1}})$$. Hence

$$\begin{aligned} P_{\overline{n}} = P_n \frac{2 \sin \left( \pi \left( \frac{1}{2^{k+2}} + \frac{3}{2^{s+1}}\right) \right) \cos \left( \pi \frac{3}{2^{s-k+1}}\right) }{\sin \left( \pi \left( \frac{1}{2^{k+1}} + \frac{3}{2^{s+1}}\right) \right) }. \end{aligned}$$

Here $s \ge 4$ and $1 \le k\le s-3$. Some tedious but elementary analysis of the function

$$\begin{aligned} g(x,y) := \frac{2\sin \left( \pi \left( \frac{x}{4} + \frac{3}{2} y\right) \right) \cos \left( \pi \frac{3}{2} \frac{y}{x}\right) }{\sin \left( \pi \left( \frac{x}{2} + \frac{3}{2} y\right) \right) } \end{aligned}$$

for $0 < y \le \frac{1}{16}$ and $8 y \le x\le \frac{1}{2}$ shows that $g (x,y) > 1$ in this region. Hence $P_{\overline{n}} > P_n$. $\square$

### Proof of Theorem 5

Consider *n* with $2^{s} \le n < 2^{s+1}$. From Lemma 6 and Lemma 7 it follows that for $2^{s}+2^{s-1} \le n < 2^{s+1}$ the product $P_n$ has its largest values for

$$\begin{aligned} n_{1}= & {} 111\ldots 11110=2^{s+1}-2\\ n_{2}= & {} 111\ldots 11101=2^{s+1}-3\\ n_{3}= & {} 111 \ldots 11100=2^{s+1}-4 \end{aligned}$$

and for $2^{s} \le n < 2^{s}+2^{s-1}$ the product $P_n$ has its largest values for

$$\begin{aligned} n_{4}= & {} 101\ldots 11110=2^{s+1}-2^{s-1}-2\\ n_{5}= & {} 101 \ldots 11101=2^{s+1}-2^{s-1}-3\\ n_{6}= & {} 101\ldots 11100=2^{s+1}-2^{s-1}-4. \end{aligned}$$

By equation (i) of Lemma 3 we have

$$\begin{aligned} P_{n_{1}} = \frac{2^s}{\sin \left( \pi /2^{s+1}\right) } \end{aligned}$$

hence $\frac{1}{n_1^2} P_{n_{1}} \rightarrow \frac{1}{2 \pi }$ for *s* to infinity. Furthermore,

$$\begin{aligned} P_{n_2}&= \frac{2^s}{\sin \left( \pi /2^{s+1}\right) f\left( 2^{s+1}-2\right) } = \frac{2^s}{\sin \left( \pi /2^{s+1}\right) 2 \sin \left( \pi \left( \frac{1}{2} - \frac{1}{2^{s+1}}\right) \right) } \\&= \frac{2^{s-1}}{\sin \left( \pi /2^{s+1}\right) \cos \left( \pi /2^{s+1}\right) }, \end{aligned}$$

and hence $\frac{1}{n_2^2} P_{n_{2}} \rightarrow \frac{1}{4 \pi }$ for *s* to infinity. Finally

$$\begin{aligned} P_{n_3}= & {} \frac{2^{s-1}}{\sin \left( \pi /2^{s+1}\right) \cos \left( \pi /2^{s+1}\right) f\left( 2^{s+1}-3\right) } \\= & {} \frac{2^{s-1}}{\sin \left( \pi /2^{s+1}\right) \cos \left( \pi /2^{s+1}\right) 2\sin \left( \pi \left( 1-\frac{1}{4}-\frac{1}{2^{s+1}}\right) \right) } \\= & {} \frac{2^{s-2}}{\sin \left( \pi /2^{s+1}\right) \cos \left( \pi /2^{s+1}\right) \sin \left( \pi \left( \frac{1}{4} + \frac{1}{2^{s+1}}\right) \right) }. \end{aligned}$$

Let now $2^{s}+2^{s-1}\le n \le n_{3}$ be arbitrary. Then

$$\begin{aligned} \frac{1}{n^{2}} P_n \le \frac{1}{\left( 2^{s}+2^{s-1}\right) ^2} P_{n_3}, \end{aligned}$$

and the last term tends to

$$\begin{aligned} \frac{2}{9 \pi \sin \frac{\pi }{4}} < \frac{1}{2 \pi }. \end{aligned}$$

Hence for all *s* large enough we have $\frac{1}{n^{2}} P_n < \frac{1}{2 \pi }$ for all $2^{s} + 2^{s-1} \le n < n_{3}$.

We still have to consider *n* with $2^{s} \le n < 2^{s}+2^{s-1}$. With equation (ii) of Lemma 3 we have

$$\begin{aligned} P_{n_4}&= P_{n_1} \frac{1}{f(1011\ldots 111) \prod ^{2^{s-1}-2}_{i=0} f(11000\ldots 00+i)} \\&= P_{n_1} \frac{1}{2 \sin \left( \frac{3\pi }{2} \frac{1}{2^{s}}\right) } \frac{\sin \left( \frac{\pi }{2^{s+1}}\right) }{\sin \frac{3 \pi }{4}}. \end{aligned}$$

The product $\kappa _{s}$ of the last two factors tends to $\frac{1}{3 \sqrt{2}}$ for *s* to infinity.

Furthermore, it is easily checked that $P_{n_5}$ and $P_{n_6}$ are smaller than $P_{n_4}$. Hence for all *n* with $2^s \le n < 2^s + 2^{s-1}$ we have

$$\begin{aligned} \frac{P_n}{n^2} \le \frac{P_{n_4}}{2^{2s}} = \frac{P_{n_1}}{n_1^2} \frac{(2^{s+1}-2)^2}{2^{2s}} \kappa _s \end{aligned}$$

which tends to $\frac{1}{2 \pi } \frac{4}{3 \sqrt{2}} < \frac{1}{2 \pi }$ for *s* to infinity. So altogether we have shown that

$$\begin{aligned} \limsup _{n \rightarrow \infty } \frac{1}{n^{2}} \prod ^{n}_{i=1} 2 \sin (\pi x_i) = \frac{1}{2 \pi }. \end{aligned}$$

From Lemma 6 and from equation (i) of Lemma 3 it also follows that for all *s* we have

$$\begin{aligned} \min _{2^s \le n < 2^{s+1}} P_{n} = P_{2^s} = 2^{s+1} \sin \left( \frac{\pi }{2^{s+1}}\right) \end{aligned}$$

which tends to $\pi$ for *s* to infinity. This gives the lower bound in Theorem 5. $\square$

## Proof of the probabilistic results

In the first part of this section we consider products

11 $$\begin{aligned} P_{N}=\prod ^{N}_{k=1}2\sin (\pi X_{k}), \end{aligned}$$

where $(X_{k})_{k\ge 1}$ is a sequence of i.i.d. random variables on [0, 1]. We want to determine the almost sure asymptotic behavior of (11). We take logarithms and define

12 $$\begin{aligned} S_{N}=\log P_{N}=\sum ^{N}_{k=1}\log (2\sin (\pi X_{k}))= \sum ^{N}_{k=1}Y_{k}, \end{aligned}$$

where $Y_{k}= \log (2\sin (\pi X_{k}))$ is again an i.i.d. sequence. Thus we can apply Kolmogorov’s law of the iterated logarithm [27] (see also Feller [12]) in the i.i.d. case. However, for later use we state this LIL in a more general form below.

### Lemma 8

Let $(Z_{k})_{k\ge 1}$ be a sequence of independent random variables with expectations $\mathbb {E}Z_{k}= 0$ and finite variances $\mathbb {E}Z^{2}_{k}<\infty$, and let $B_{N}=\sum ^{N}_{k=1}\mathbb {E}Z_{k}^{2}$. Assume there are positive numbers $M_{N}$ such that

13 $$\begin{aligned} |Z_{N}|\le M_{N} \quad \text {and} \quad M_{N}=o\left( \sqrt{\frac{B_{N}}{\log \log B_{N}}}\right) . \end{aligned}$$

Then $S_{N}=\sum ^{N}_{k=1}Z_{k}$ satisfies a law of the iterated logarithm

14 $$\begin{aligned} \limsup _{N\rightarrow \infty }\frac{S_{N}}{\sqrt{B_{N}\log \log B_{N}}}= \sqrt{2} \qquad \text {almost surely.} \end{aligned}$$

In the case of centered i.i.d. random variables ${Z}_{k}$ with finite variance, we have $B_{N}=bN$ with $b=\mathbb {E}Z^{2}_{1}$. Thus in this case

15 $$\begin{aligned} \limsup _{N\rightarrow \infty }\frac{S_{N}}{\sqrt{N \log \log N}}=\sqrt{2 b}\quad \quad \quad \text {almost surely.} \end{aligned}$$

In order to apply Lemma 8 to the sum (12), we note that

$$\begin{aligned} \mathbb {E} Y_k = \mathbb {E} (\log (2\sin (\pi X_{k}))) = \int ^{1}_{0}\log (2 \sin (\pi x)) \,\mathrm{d}x= 0, \end{aligned}$$

and compute the variance

$$\begin{aligned} \mathbb {E} Y_k^ 2 = \mathbb {E} (\log ^{2}(2\sin (\pi X_{k}))) =\int ^{1}_{0}\log ^{2}(2\sin (\pi x)) \,\mathrm{d}x=\frac{\pi ^2}{12}. \end{aligned}$$

This proves Theorem 6.

For the proof of Theorem 7 we split the corresponding logarithmic sum into two parts

16 $$\begin{aligned}&\sum _{1 \le n_k \le N} \log (2\sin (\pi n_{k}\alpha )) \nonumber \\&\quad = \frac{1}{2} \left( \sum ^{N}_{n=1}\log (2\sin (\pi n \alpha )) + \sum ^{N}_{n=1} R_{n}\log (2\sin (\pi n\alpha )) \right) , \end{aligned}$$

where $R_{n}=R_{n}(t)$ denotes the $n{\mathrm{th}}$ Rademacher function on [0, 1] and the space of subsequences of the positive integers corresponds to [0, 1] equipped with the Lebesgue measure. For irrationals $\alpha$ with bounded continued fraction expansion, by Corollary 1 we have

17 $$\begin{aligned} \sum ^{N}_{n=1}\log (2\sin (\pi n \alpha )) =O(\log ^{2}N). \end{aligned}$$

For the second sum in (16) we set $Z_{n}=R_{n}\log (2 \sin (\pi n \alpha ))$ and apply Lemma 8. The random variables $Z_{n}$ are clearly independent and thus we have to compute the quantities $B_{N}$ and check condition (13). Obviously, $\mathbb {E}Z_{n}=0$ and $\mathbb {E}Z^{2}_{n}=\log ^{2} (2\sin (\pi n \alpha ))$. Using the fact that

$$\begin{aligned} |\sin (\pi n \alpha )|\ge 2 \Vert n \alpha \Vert \ge \frac{{c}_{0}}{n}, \end{aligned}$$

with some positive constant $c_0$, we obtain

$$\begin{aligned} | Z_{N}| \le c_{1} \log N \end{aligned}$$

with some $c_{1}> 0$. Using Koksma’s inequality and discrepancy estimates for $(n \alpha )_{n \ge 1}$ it can easily been shown that

$$\begin{aligned} \frac{B_{N}}{N} = \frac{1}{N}\sum _{n=1}^N \log ^{2} (2\sin (\pi n \alpha )) \rightarrow \int _0^1 \log ^{2} (2\sin (\pi n \alpha )) \,\mathrm{d}\alpha = \frac{\pi ^2}{12}. \end{aligned}$$

Thus, the conditions of Lemma 8 are satisfied and we have

$$\begin{aligned} \limsup _{N \rightarrow \infty } \frac{\sum _{n=1}^N Y_n}{\sqrt{N \log \log N}} = \frac{\pi }{\sqrt{6}},\quad \mathbb {P}\text {-almost surely.} \end{aligned}$$

Consequently, from (16) and (17) we obtain

18 $$\begin{aligned} \limsup _{N \rightarrow \infty } \frac{\sum _{1 \le n_k \le N} \log (2\sin (\pi n_{k}\alpha ))}{\sqrt{N \log \log N}} = \frac{\pi }{2\sqrt{6}}, \quad \mathbb {P}\text {-almost surely.} \end{aligned}$$

Finally, note that by the strong law of large numbers we have, $\mathbb {P}$-almost surely, that

$$\begin{aligned} \# \left\{ k:~1 \le n_k \le N\right\} \sim \frac{N}{2}. \end{aligned}$$

Consequently, from (18) we can deduce that

$$\begin{aligned} \limsup _{N \rightarrow \infty } \frac{\sum _{k=1}^N \log (2\sin (\pi n_{k}\alpha ))}{\sqrt{N \log \log N}} = \frac{\pi }{\sqrt{12}}, \quad \mathbb {P}\text {-almost surely.} \end{aligned}$$

This proves Theorem 7.

## Footnotes

1 The second paper was published in a seminar proceedings volume called “Zahlentheoretische Analysis”. Hlawka introduced this term for applications of number-theoretic methods in real or complex analysis. In particular, he often applied uniformly distributed sequences to give discrete versions of continuous models.
